# Clinical Implications of 20-Hydroxyeicosatetraenoic Acid in the Kidney, Liver, Lung and Brain: An Emerging Therapeutic Target

**DOI:** 10.3390/pharmaceutics9010009

**Published:** 2017-02-20

**Authors:** Osama H. Elshenawy, Sherif M. Shoieb, Anwar Mohamed, Ayman O.S. El-Kadi

**Affiliations:** 1 Faculty of Pharmacy and Pharmaceutical Sciences, University of Alberta, Edmonton T6G 2E1, AB, Canada; oshenawy@ualberta.ca (O.H.E.); shoieb@ualberta.ca (S.M.S.); anwarmoh@ualberta.ca (A.M.); 2 Department of Basic Medical Sciences, College of Medicine, Mohammed Bin Rashid University of Medicine and Health Sciences, Dubai, United Arab Emirates

**Keywords:** 20-hydroxyeicosatetraenoic acid (20-HETE), Cytochrome P450s (CYPs), arachidonic acid (AA), kidney, ischemia/reperfusion (I/R) injury, liver, lung, brain

## Abstract

Cytochrome P450-mediated metabolism of arachidonic acid (AA) is an important pathway for the formation of eicosanoids. The ω-hydroxylation of AA generates significant levels of 20-hydroxyeicosatetraenoic acid (20-HETE) in various tissues. In the current review, we discussed the role of 20-HETE in the kidney, liver, lung, and brain during physiological and pathophysiological states. Moreover, we discussed the role of 20-HETE in tumor formation, metabolic syndrome and diabetes. In the kidney, 20-HETE is involved in modulation of preglomerular vascular tone and tubular ion transport. Furthermore, 20-HETE is involved in renal ischemia/reperfusion (I/R) injury and polycystic kidney diseases. The role of 20-HETE in the liver is not clearly understood although it represents 50%–75% of liver CYP-dependent AA metabolism, and it is associated with liver cirrhotic ascites. In the respiratory system, 20-HETE plays a role in pulmonary cell survival, pulmonary vascular tone and tone of the airways. As for the brain, 20-HETE is involved in cerebral I/R injury. Moreover, 20-HETE has angiogenic and mitogenic properties and thus helps in tumor promotion. Several inhibitors and inducers of the synthesis of 20-HETE as well as 20-HETE analogues and antagonists are recently available and could be promising therapeutic options for the treatment of many disease states in the future.

## 1. Introduction

**Figure 1:**
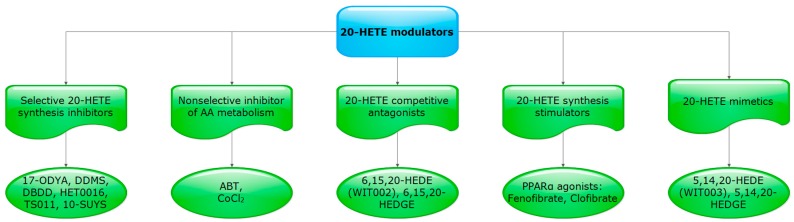
Different 20-hydroxyeicosatetraenoic acid (20-HETE) modulators commonly used to study the role of 20-HETE in vivo and in vitro.

Arachidonic acid (AA), which is a major component of cell membrane, is known to be metabolized into different classes of eicosanoids, by cyclooxygenase (COX), lipoxygenase (LOX), and cytochrome P450 (CYP). COX is known to be responsible for production of prostaglandins (PGs); whereas LOX produces mid chain hydroxyeicosatetraenoic acids (HETEs), lipoxins (LXs), and leukotrienes (LTs). CYP enzymes produce epoxyeicosatrienoic acids (EETs) by CYP epoxygenases, and HETEs (terminal, sub-terminal, and mid-chain) by CYP hydroxylases [1,2,3,4]. Terminal hydroxylation of AA is known as ω-hydroxylation reaction in which AA is converted to 20-HETE through CYP4A and CYP4F enzymes [5,6,7]. COX plays an important role in metabolism of 20-HETE providing a diverse range of activities in different organs [8]. 20-HETE is metabolized by COX into hydroxyl analogue of vasoconstrictor prostaglandin H2 (20-OH PGH_2_) which is further transformed by isomerases into vasodilator/diuretic metabolites (20-OH PGE_2_, 20-OH PGI_2_) and vasoconstrictor/antidiuretic metabolites (20-OH Thromboxane A_2_, 20-OH PGF_2a_) [9,10,11]. A number of selective inhibitors for 20-HETE synthesis have been previously used including 17-octadecynoic acid (17-ODYA), *N*-methylsulfonyl-12,12-dibromododec-11-enamide (DDMS), dibromododec-11-enoic acid (DBDD), *N*-hydroxy-*N’*-(4-butyl-2methylphenyl)formamidine (HET0016), *N*-(3-Chloro-4-morpholin-4-yl)Phenyl-*N’*-hydroxyimido formamide (TS011) and acetylenic fatty acid sodium 10-undecynyl sulfate (10-SUYS) [5,6,12,13,14,15,16]. Nonselective inhibitors of AA metabolism were also used including 1-Aminobenzotriazole (ABT) and Cobalt (II) chloride (CoCl_2_) [17,18]. Recently, competitive antagonists have been employed including 20-hydroxyeicosa-6(Z),15(Z)-dienoic acid (6,15,20-HEDE; WIT002) and 20-hydroxyeicosa-6(Z),15(Z)-dienoyl]glycine (6,15,20-HEDGE) [5,13,14,15]. Peroxisome proliferator-activated receptor alpha (PPARα) agonists, such as fenofibrate and clofibrate, or gene therapy were used to upregulate the formation of 20-HETE besides 20-HETE mimetics, 20-hydroxyeicosa-5(Z),14(Z)-dienoic acid (5,14,20-HEDE; WIT003), and *N*-[20-hydroxyeicosa-5(Z),14(Z)-dienoyl]glycine (5,14,20-HEDGE) [13,15] (Figure 1 represents a summarization for 20-HETE modulators commonly used in previous literature).

**Figure 2:**
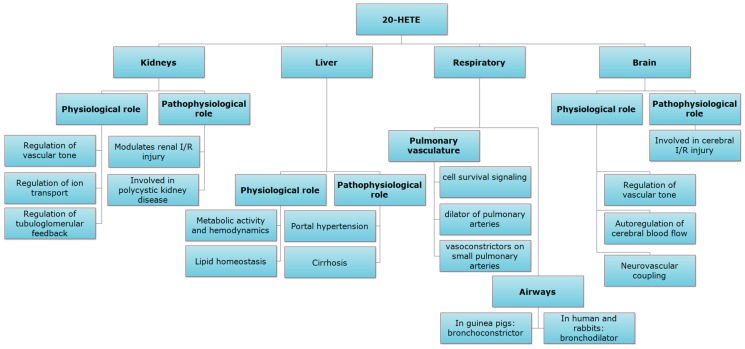
Role of 20-HETE in the kidney, liver, lung and brain during normal physiological and pathophysiological conditions.

Notably, eicosanoids exert their action through specific receptors called eicosanoid receptors, in addition to non-specific receptors such as PPAR receptors [19]. Recent data demonstrated the identification of a novel G protein-coupled receptor (GPCR) as 20-HETE receptor in the vascular endothelium [20]. The identification of 20-HETE receptor would result in better understanding of molecular mechanisms and clinical implications of 20-HETE in different organs. In this review, 20-HETE role in the kidney, liver, lung and brain during normal physiology, and during pathophysiological disease states will be discussed (summarized in Figure 2).

Moreover, we will discuss 20-HETE role in mitogenicity. Furthermore, we will discuss the possible therapeutic approaches using 20-HETE mimetics, antagonists as well as synthesis inducers and inhibitors.

## 2. Role of 20-HETE in the Kidney

The kidney has the highest abundance of CYP among all extrahepatic organs, and the highest level within the kidney was found in the proximal tubules [21,22]. 20-HETE was identified as the major CYP metabolite of AA in the proximal tubule [21] and microsomes of renal cortex [23]. In thick ascending limb of the loop of Henle (TAL), 20-HETE and 20-carboxyeicosatetraenoic acid (20-COOH-AA) are the major AA metabolites of the CYP-dependent pathway [24,25]. 20-HETE is also a major AA metabolite in the renal microvasculature [26,27,28] and acts as a potent vasoconstrictor; however, its vasoconstrictor actions can be offset by its natriuretic properties [29]. 20-HETE contracts renal microvessels at concentrations of less than 10^−10^ M [30] and sensitizes renal vessels transfected with CYP4A1 cDNA to phenylephrine [31,32]. Also there is a strong evidence that locally produced 20-HETE plays a pivotal role in modulating the myogenic responsiveness of the afferent arteriole and may help explain how deficiencies in the renal production of 20-HETE could foster the initiation of hypertension-induced glomerular injury [33]. Therefore, 20-HETE is the preeminent renal eicosanoid, overshadowing PGE_2_ and PGI_2_ [8] and plays a role in vascular and tubular abnormalities of renovascular disease states [34]. Interestingly, 20-HETE reduces albumin permeability (P_alb_), while on the other hand its relatively lowered levels are associated with increased P_alb_, development of proteinuria and glomerular injury in early hypertension. Pretreatment of Sprague Dawley (SD) rats glomeruli with the 20-HETE mimetic, 5,14,20-HEDE, reduced baseline P_alb_ and opposed the effects of transforming growth factor-beta (TGF-β) to increase P_alb_ [35,36,37]. Moreover, exogenous 20-HETE or clofibrate treatment protected the glomeruli from increased P_alb_ caused by puromycin aminonucleoside, which is known to be an injurious agent [36].

### 2.1. Biosynthesis of 20-HETE in the Kidney

**Figure 3:**
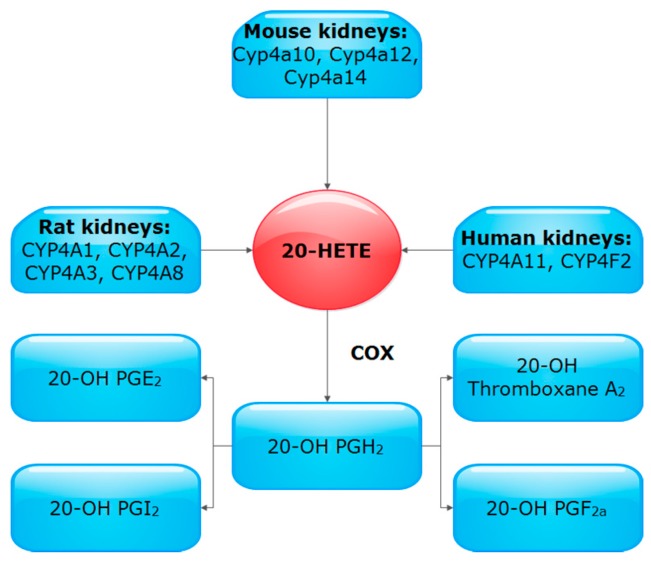
Enzymes responsible for 20-HETE formation and metabolism in different species.

20-HETE production in the kidney has been extensively studied in rats, mice, and humans. In this regard, it was found that 20-HETE is formed primarily by CYP4A and CYP4F subfamilies (Figure 3) [5,38].

In rat kidney, different CYP4A isoforms were detected, namely CYP4A1, CYP4A2, CYP4A3 and CYP4A8 [26,27,39,40,41]. Each of these isozymes contribute to a different extent to the basal renal function [42]. For example, CYP4A1 is characterized as a major 20-HETE synthesizing isozyme in the rat kidney [27,43,44]. On the other hand, CYP4A2 is a major contributor to hemodynamic responses, whereas CYP4A3 is a major contributor to tubular responses following nitric oxide (NO) inhibition [42]. CYPA expression and 20-HETE synthesis were the highest in the outer medulla followed by the cortex and lastly the inner medulla/papilla [45]. Also in rats, different CYP4F isoforms have been detected with CYP4F1, CYP4F4, and CYP4F5 being more expressed in the renal cortex than the medulla, while CYP4F6 shows higher medullary expression [46]. In the mouse, however, Cyp4a10, Cyp4a12, and Cyp4a14 are involved in 20-HETE synthesis, of which Cyp4a12a is the predominant 20-HETE synthase [47]. Interestingly, Cyp4a12a expression determines the sex and strain specific differences in 20-HETE generation [48]. Microdissected renal blood vessels and nephron segments from C57BL/6J mice revealed that Cyp4a and Cyp4f isoforms were detected in every segment analyzed [38]. In humans, it was found that microsomes from kidney cortex, converted AA mainly to 20-HETE by CYP4A11 and CYP4F2 [49,50]. Of interest, different human CYP4F2 variants have been identified, of which M433 allele was found to be associated with 56%–66% decrease in 20-HETE production [51].

### 2.2. Metabolism and Regulation of 20-HETE in the Kidney

Metabolism of 20-HETE by COX (Figure 3) is proposed to represent an important regulatory mechanism in setting preglomerular microvascular tone [52]. Renal vasoconstrictive effect of 20-HETE in response to hyperchloremia was shown to be dependent on COX activity [11]. Also, low-salt intake was found to stimulate the renin-angiotensin system and induces renal vascular expression of CYP4A and COX-2 in arcuate and interlobular arteries while COX-1 was unaffected [52]. It was found that low-salt diet increases 20-HETE levels in the incubate of either arcuate/interlobular or interlobular renal arteries only when COX was inhibited [52]. Thus, the capacity of COX to metabolize 20-HETE to PG analogs, e.g., 20-OH PGF_2a_ and 20-OH PGE_2_, may be critical to modify the renal vascular and tubular actions of the eicosanoids [53].

With regards to regulation of 20-HETE in the kidney, inhibition of its formation contributes to the cGMP-independent vasodilator response to NO in the renal microcirculation [8,54,55,56,57,58], attenuates myogenic tone and autoregulation of blood flow, and modulates vascular responses to the vasodilators, such as carbon monoxide (CO), and vasoconstrictors such as angiotensin II (AngII) and endothelin (ET) [59]. Interestingly, NO inhibits a variety of heme-containing enzymes, including NO synthase (NOS) and CYP enzymes. For example, it inhibits CYP4A exerting a negative modulatory effect on 20-HETE formation. Inhibition of NOS was found to increase ω-hydroxylase activity, CYP4A expression, and renal efflux of 20-HETE with a concomitant enhanced response to vasoconstrictor agents [8,54,55,56,57,58]. Inhibition of NOS with N(ω)-nitro-l-arginine-methyl ester (l-NAME) greatly increased the expression of ω-hydroxylase protein and induced a 4-fold increase in renal efflux of 20-HETE [55]. In addition, l-NAME increased mean arterial blood pressure and renal vascular resistance (RVR), while reducing renal blood flow and GFR associated with diuresis and natriuresis. Importantly, DBDD, as a 20-HETE synthesis inhibitor, was able to blunt these effects [10]. Sodium nitroprusside, a NO donor, inhibited renal microsomal conversion of AA to 20-HETE and increased vascular diameter in a dose-dependent manner [54,55,57].

Heme and products derived from its metabolism, by heme oxygenase enzymes (HO-1 and HO-2), were found to influence renal function and blood pressure potentially by affecting the expression and activity of hemoproteins, including CYP and COX isoenzymes (COX-1 and COX-2) [45]. HO isoform expression was found to be segmented within the kidney and along the nephron [45]. HO-1 protein in kidney was barely detectable; its contribution to the regulation of hemoproteins became apparent only under pathophysiological conditions that caused HO induction. To the contrast, HO-2 protein was found to be expressed in all kidney structures with higher levels in outer medulla followed by inner medulla/papilla and cortex [45,60]. HO-1 induction was found to suppress microsomal heme, CYP4A and COX-2 protein, and 20 HETE [45,60].

Treatment with clofibrate, a PPAR_α_ agonist, increased CYP4A protein levels and the subsequent 20-HETE production in microsomes prepared from the renal cortex [61,62]. Moreover, in an in vitro study performed in human renal tubular epithelial cells (HK-2 cell line), Li et al. have demonstrated that cisplatin is a potent inducer of CYP4A11 and it exerts its cytotoxic activity in kidney via increasing production of 20-HETE [63]. In contrast, treatment with pioglitazone, the PPAR_γ_ agonist, neither affected CYP4A nor 20-HETE level [61,62]. Similarly, dexamethasone, an inducer of CYP4A, caused a 2-fold increase in the proximal tubular synthesis of 20-HETE as well as an increase in CYP4A1 mRNA [61]. Another regulator for 20-HETE production is the dietary salt intake which modulates glomerular CYP activity [57]. Hyperchloremia increased 20-HETE release from the rat kidney by 2-fold when compared with low-chloride conditions of renal perfusion [11]. As for AngII, it was found to induce 20-HETE release from preglomerular microvessels [52], whereas vasopressin deficiency elevates the expression of CYP4A protein and renal formation of 20-HETE in the kidney of Brattleboro rats [64].

### 2.3. Role of 20-HETE in the Kidney

#### 2.3.1. Role of 20-HETE in Preglomerular Vascular Tone Regulation

CYP4A isozymes have the potential to function as an oxygen sensor in mammalian microcirculatory beds and to regulate arteriolar tone by generating 20-HETE in an oxygen-dependent manner [40]. 20-HETE is produced by renal vascular smooth muscle (VSM) cells [65] and participates in the autoregulation of renal blood flow via its effect on vascular tone, ion transport and tubuloglomerular feedback; while excess 20-HETE was found to be excreted in the urine [8,66,67,68,69]. Many previous studies have addressed 20-HETE as a second messenger that plays a central role in the regulation of renal vascular tone [38,41,47,48,61,70]. 20-HETE is a potent constrictor produced by the preglomerular vasculature; it contributes to the regulation of tone and myogenic response [40,43,56,71,72]. In addition, 20-HETE contributes to the increase in intracellular calcium in renal micro-VSM cells [73]. The vasoconstrictor response of canine renal arcuate arteries to 20-HETE resembles the myogenic activation of these vessels after elevations in transmural pressure [74].

**Figure 4:**
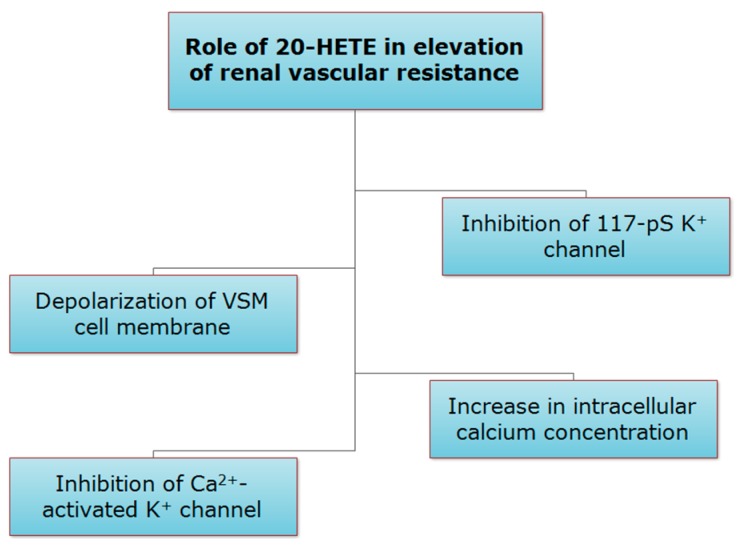
Role of 20-HETE in elevation of renal vascular resistance.

20-HETE contributes importantly to renal blood flow autoregulation by afferent arterioles [28], and contributes to the development of hypertension by elevating renal vascular resistance [75]. Conversely, in the kidney tubules, 20-HETE suppresses sodium reabsorption and enhances natriuresis, hence, contributing to antihypertensive mechanisms [76]. 20-HETE acts in part by inhibiting the opening of the large-conductance Ca^2+^-activated K^+^ channel, depolarizing VSM cell membrane, and producing sustained increase in intracellular calcium concentration [8,28,59,65,77,78,79,80,81]. Inhibition of 20-HETE formation contributes to the activation of K^+^ channels and the vasodilator effects of NO in the renal microcirculation [82]. As for renal medullary perfusion, it seems to be under tonic suppression by 20-HETE, where renal medullary perfusion indices were increased after infusion of HET0016 [83]. Similarly, infusion of 17-ODYA into the renal artery increased cortical and papillary blood flows [66]. In isolated canine renal arcuate arteries, 20-HETE significantly reduced mean open time, the open-state probability, and the frequency of opening a 117-pS K^+^ channel recorded from renal VSM cells (Figure 4) [74].

Afferent arteriolar vasoconstriction is partly mediated by the endothelial COX pathway. 20-HETE caused constriction of rabbit afferent arterioles when vascular tone increased with norepinephrine. However, this constriction was significantly attenuated by pretreatment with indomethacin or the thromboxane/endoperoxide receptor antagonist, SQ29548 [75]. 20-HETE metabolites, 20-OH PGE_2_ and 20-OH PGF_2α_ play significant role in regulating cortical blood flow and medullary blood flow. Renal intra-arterial infusion of 20-HETE was found to decrease cortical blood flow and increase medullary blood flow. These controversial effects were attributable to 20-OH PGF_2α_ that caused vasoconstriction, and 20-OH PGE_2_ that caused vasodilation in the cortex and medulla [84]. 20-HETE is a second messenger for ET-1 and a mediator for selective renal effects of AngII [8]. ET-1 increased mean arterial blood pressure, renal vascular resistance, while reduced cortical blood flow and renal blood flow, at least in part, by modulating 20-HETE levels via activation of ET receptors [84,85,86,87]. However, ET-1 also increased medullary blood flow which seemed to be mediated by the vasodilator prostanoids or by NO via ET receptor activation [85]. As for transgenic animals, KAP-CYP4F2 transgenic mice, predominantly showed renal overexpression of CYP4F2 leading to high 20-HETE in urine and blood, and accounting for the elevation in blood pressure [88]. Cyp4a14 KO mice have prohypertensive status as a result of increased Cyp4a12 expression with associated increased production of 20-HETE and endothelial NOS (eNOS) uncoupling leading to increased oxidative stress, enhanced vasoconstriction as well as a defect in the renal excretory capacity [47]. Recently, it was shown that the dysregulated renal 20‑HETE/EET ratio in the hypertensive Cyp4F2 transgenic mice was resulted from the activation of soluble epoxide hydrolase (sEH) and the inhibition of epoxygenase activity suggesting 20-HETE vasoconstrictive activity. Moreover, 20‑HETE demonstrated an inverse regulatory effect on the endogenous epoxygenases in the kidney [89].

#### 2.3.2. Role of 20-HETE in Tubular Ion Transport

**Figure 5:**
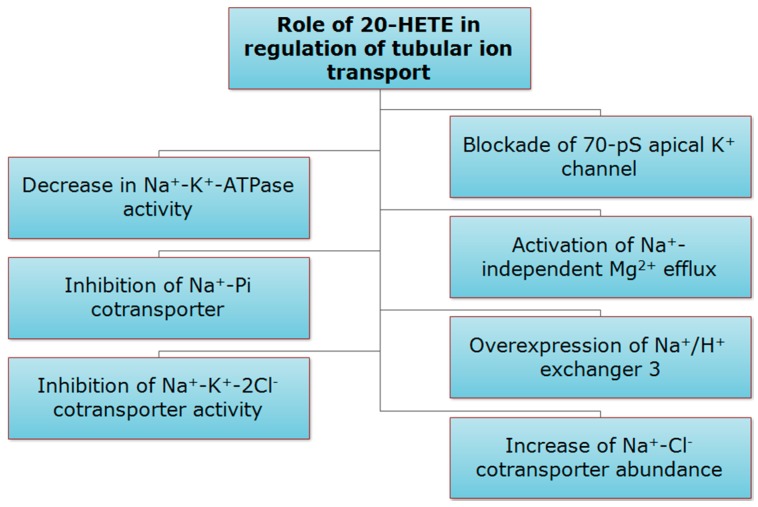
Role of 20-HETE in regulation of tubular ion transport.

20-HETE serves as a second messenger that plays a central role in the regulation of sodium reabsorption in the proximal tubule and TAL [21,38,41,47,48,61,70]. 20-HETE inhibits sodium-potassium-chloride (Na^+^-K^+^-2Cl^−^) transporter in the TAL [90]. Moreover, 20-HETE, produced in the proximal tubules, modulates ion transport by regulating Na^+^-K^+^-ATPase activity via stimulating protein kinase C (PKC) to phosphorylate the α-subunit of Na^+^-K^+^-ATPase [8,28,59,65,91,92,93,94,95]. Additionally, excessive fluid reabsorption in the proximal tubule in the Cyp4a14 knockout mice has led to hypertension which is secondary to 20-HETE-mediated overexpression of sodium-hydrogen exchanger 3 [96,97,98]. Infusion of 20-HETE at a dose of 100 nmol/kg/min decreased medullary Na^+^-K^+^-ATPase activity by 24.2% [99]. 20-HETE serves also as a second messenger for the natriuretic effects of dopamine, parathyroid hormone, and AngII [65,100,101,102]. In addition, 20-HETE specifically inhibits Na^+^-Pi co-transporter in proximal tubule-like opossum kidney (OK) cells [103,104,105,106]. As for TAL, 20-HETE modulates ion transport by inhibiting the activities of Na^+^-K^+^-2Cl^−^ co-transporter [8,28,65,93,94,107,108]. 20-HETE inhibits Na^+^-K^+^-2Cl^−^ transport, in part, by blocking a 70-pS apical K^+^ channel, the predominant type of the two apical K^+^ channels operating under physiological conditions in the TAL of the rat kidney [59,109,110]. DDMS was found to increase the activity of the 70-pS K^+^ channel significantly [111]. Interestingly, AngII has dual effects on the activity of the apical 70-pS K^+^ channel in TAL of the rat kidney, where 50 pM AngII has inhibitory effect mediated by 20-HETE, whereas 50–100 nM AngII has stimulatory effect mediated via NO [24]. In addition, 20-HETE was found to be a key mediator in the activation of Na^+^-independent Mg^2+^ efflux (Figure 5) [112]. Recently, it has been shown that human CYP4A11 transgenic mice have developed a 20-HETE-dependent hypertension associated with 50% increase in sodium chloride cotransporter abundance in the distal tubules [113]. 

### 2.4. Role of 20-HETE in Pathophysiology of the Kidney

#### 2.4.1. Role of 20-HETE in Renal Ischemia/Reperfusion (I/R) Injury

Acute kidney injury (AKI) is a major consequence following surgery and various medical conditions that significantly increases morbidity and mortality [114,115,116]. Recent reports suggest that renal I/R injury is the most common cause of AKI [50]. Several reports showed that AA is released from membrane phospholipids in response to ischemia and can be metabolized to 20-HETE that plays an important role in I/R injury which is known to be associated with a patient mortality of up to 50% [117,118]. 20-HETE overexpression in renal I/R injury exacerbates cellular damage by enhancing generation of free radicals and activation of caspase-3 [119]. 20-HETE role in vasoconstriction and inflammation contributes to I/R injury as well [120]. 20-HETE also plays a role in post-ischemic fall in medullary blood flow and its associated long-term decline in renal function [121]. In this regard, the 20-HETE antagonist, 6,15,20-HEDE, accelerated the recovery of medullary perfusion as well as renal medullary and cortical re-oxygenation, during early reperfusion phase, whereas the 20-HETE mimetic, 5,14,20-HEDE, did not improve renal injury and reversed the beneficial effects of 6,15,20-HEDE [120]. Pretreatment of uninephrectomized male Lewis rats with either the HET0016 or 6,15,20-HEDE via the renal artery before exposure to warm ischemia was found to attenuate I/R induced renal dysfunction and to reduce tubular lesion scores, inflammatory cell infiltration, and tubular epithelial cell apoptosis [120]. However contradicting results were obtained in a previous study in which infusion of 5,14-20-HEDE or 5,14-20-HEDGE protected SD rat kidney from I/R injury, whereas HET0016 exacerbated renal injury [15]. Protection provided by 20-HETE analogues was supposed to be due to inhibition of renal tubular sodium transport, increased urine output and sodium excretion, and prevention of post-ischemic fall in medullary blood flow [15]. The contradiction in results could be related to the use of different models and treatment forms. In addition, high systemic HET0016 levels may inhibit not only 20-HETE synthesis, but also the ω-hydroxylation and inactivation of leukotriene B4 resulting in aggravation of ischemic renal damage. Moreover, high doses of 5,14-20-HEDE (mg/kg range) results in high systemic drug levels that acted not only during ischemia but also during reperfusion providing protection for the kidney mainly in reperfusion phase by inhibiting sodium reabsorption, thereby limiting tubular oxygen consumption [120]. In renal transplant patients, the change in dynamics of 20-HETE during early phase of allograft reperfusion was associated with early post-transplant graft function, and extent of 20-HETE release occurring within the first 5 min of allograft reperfusion was found to be a negative predictor of post-transplant allograft function [117,120].

#### 2.4.2. Role of 20-HETE in Polycystic Kidney Diseases

Polycystic kidney diseases are characterized by abnormal proliferation of renal epithelial cells which was found to be mediated by 20-HETE [122,123,124]. Several pathways known to be involved in polycystic kidney disease have been reported to be activated by 20-HETE such as PKC, Src and EGFR pathways. Expression of CYP4A1, CYP4A2, CYP4A3, and CYP4A8 mRNA was found to be increased in polycystic kidney by 2- to 4-fold as compared to non-cystic SD rat kidney; and daily administration of HET0016 significantly reduced kidney size by 24% [123]. Similarly, Balb/c polycystic kidney mouse, a model of autosomal recessive polycystic kidney disease, treated with HET0016 daily showed significant reduction in kidney size by half and approximately doubled survival rates [122]. Non-cystic Balb/c cells overproducing Cyp4a12 exhibited a 4- to 5-fold increase in cell proliferation compared with control Balb/c cells, however this effect was completely abolished when 20-HETE synthesis was inhibited [122]. These findings suggest that 20-HETE might be a new biomarker and a therapeutic target for polycystic kidney disease [95].

### 2.5. 20-HETE Mediation of Drug Induced Toxicity in the Kidney

Tian et al. reported that 20-HETE may protect from doxorubicin-induced toxicity of human renal tubular epithelial cells. They found that doxorubicin suppresses CYP4A11 gene expression and protein production in human renal tubular epithelial cells more efficiently than HET0016. This resulted in decreased cell viability and increased lactate dehydrogenase activity when human renal tubular epithelial cells were incubated with doxorubicin for 24h, whereas 20-HETE increased cell survival and decreased lactate dehydrogenase activity in concentration-dependent manner with no reported mechanism. Similar results were obtained when 20-HETE was co-administered with doxorubicin, while, HET0016 was found to exaggerate doxorubicin effect [125]. On the other hand, 20-HETE plays a role in cyclosporin A-induced nephrotoxicity. Rats treated with cyclosporin A suffered from increased renal microsomal conversion of AA to 20-HETE, increased systolic blood pressure, and induced renal damage, whereas pretreatment with HET0016 or ABT attenuated or prevented these effects [126,127,128]. Interestingly, a recent in vitro study performed in HK-2 cells suggested that cisplatin is a potent inducer of CYP4A11 and 20-HETE biosynthesis and this mechanism of induction is proposed as a novel mechanism of renal tubular toxicity of cisplatin [63].

### 2.6. Role of 20-HETE in the Renal System during Pregnancy

20-HETE synthesis in the kidney is altered in a time- and site-specific manner during pregnancy suggesting distinct regulatory mechanisms for 20-HETE synthesis in the kidney during pregnancy [129]. In addition, NO interacts with CYP4A proteins in a distinct manner and it interferes with renal microvessels 20-HETE synthesis, emphasizing the important role of 20-HETE in regulating blood pressure and renal function during pregnancy [130,131]. Inhibition of NO synthesis by l-NAME during late pregnancy led to increased production of renal vascular 20-HETE causing significantly higher mean arterial blood pressure and renal vascular resistance, and lower renal blood flow and GFR. Combined treatment with DDMS and l-NAME significantly attenuated these effects [131]. This data suggested that 20-HETE plays a role in hypertension and renal vasoconstriction induced by chronic reduction in uterine perfusion pressure (RUPP) in pregnant rats [132]. ABT decreased mean arterial blood pressure in RUPP rats whereas it had no effect in normal pregnant rats, besides attenuating the differences in renal hemodynamics observed between normal pregnant and RUPP rats [132]. 

## 3. Role of 20-HETE in the Liver

### 3.1. Formation of 20-HETE in the Liver

Interestingly, 50%–75% of CYP-dependent AA metabolites formed by human liver microsomes are 19-HETE and 20-HETE where 20-HETE was found to be the main metabolite formed [93,133]. AA ω-hydroxylation in human liver is catalyzed by CYP4F2, CYP4F3 and CYP4A11 [134]; with CYP4F2 and CYP4F3B, human liver-specific splice variant of CYP4F3, generating most hepatic 20-HETE [134,135]. In human hepatocyte-like HepaRG, hepatoma cell line, induction of CYP4F3B was observed by PGA_1_ and by lovastatin leading to increased synthesis of 20-HETE [135,136]. In addition, statins were found to recruit PXR to induce CYP4F3B [136]. On the other hand, isoniazid was found to reduce expression and formation of both CYP4A and 20-HETE, respectively, in the liver [137].

### 3.2. Role of 20-HETE in the Liver

Very little information is available about the role of 20-HETE in the liver, however it seems that 20-HETE participates in the regulation of liver metabolic activity and hemodynamics [93,138]. 20-HETE is a potent activator of PPAR_α_ and may exert important functions in lipid homeostasis and in controlling fat-dependent energy supply and metabolism, in addition it is an important inflammatory mediator and may have important role in inflammatory diseases [135,136]. 20-HETE is a weak, COX-dependent, vasoconstrictor of the portal circulation, and it was supposed to be involved in pathophysiology of portal hypertension [93,138]. In addition, 20-HETE was found to be involved in abnormalities related to liver diseases, particularly cirrhosis [93]. While 20-HETE and PGs are excreted at similar rates in normal subjects, it was found that excretory rates of 20-HETE were several-fold higher than those of PGs and TxB_2_ in patients with cirrhosis [93,139]. Recently, 20‑HETE demonstrated a counter regulatory effect on the endogenous epoxygenases in the liver. Thus the tuning of 20-HETE and EETs formation and degradation may provide therapeutic benefits; given that they have opposite effects in several diseases [89,140]. Interestingly, 20-HETE level in the urine of cirrhotic patients with ascites is much higher than in case of cirrhosis without ascites which in turn is higher than excretion rates in normal individuals [139]. In patients with hepatic cirrhosis, 20-HETE is produced in increased amounts in the preglomerular microcirculation resulting in constriction of renal vasculature, reduction of renal blood flow and depression of renal hemodynamics [93,139]. Of interest, CYP4F2 was found to be exclusively expressed in the liver of CMV-CYP4F2 transgenic mice, driven by cyto-megalovirus (CMV) promoter, with no effect on 20-HETE levels in the urine, kidney, and blood or even on systolic blood pressure. In contrast, KAP-CYP4F2 transgenic mice, driven by kidney androgen-regulated protein (KAP) promoter, were found to overexpress renal CYP4F2 and to have high 20-HETE levels in urine and blood, in addition to elevated blood pressure [88].

## 4. Role of 20-HETE in the Respiratory System

### 4.1. Distribution of 20-HETE in Pulmonary Tissues

In the lung, 20-HETE is produced from vascular and non-vascular tissues; it was detected in pulmonary arteries, bronchi, and endothelium [141,142]. 20-HETE modulates many physiological functions such as smooth muscle tone and electrolyte flux [141] and serves as a paracrine factor in the pulmonary circulation when generated from nonvascular tissues [143]. Peripheral lung tissue, small and large pulmonary arteries, airways, and isolated VSM cells from small pulmonary arteries produced 20-HETE when incubated with AA [143]. In adult male rat lung, CYP4A mRNA was detected in pulmonary arterial endothelial and smooth muscle cells, bronchial epithelial and smooth muscle cells, type I epithelial cells, and macrophages. In addition, CYP4A protein was detected in rat pulmonary arteries and bronchi as well as cultured endothelial cells [141]. In rabbit, CYP4A is widely distributed in lung tissues as well [143]. In rats with acute pseudomonas pneumonia, 20-HETE level was depressed in microsomes prepared from lung [144], whereas in cystic fibrosis patients, 20-HETE was detected in freshly obtained sputum suggesting a role in these disease states [145].

### 4.2. Role of 20-HETE in Pulmonary Cell Survival

20-HETE mediates cell survival signaling in the pulmonary vasculature [146,147], it enhances survival and protects against apoptosis in bovine pulmonary artery endothelial cells (BPAECs) stressed by serum starvation or lipopolysaccharide [148,149]. 20-HETE enhanced survival and protected against apoptosis in mouse pulmonary arteries (PAs) exposed to hypoxia reoxygenation ex vivo [146,148], and similar results obtained with 20-HETE analogs [146]. Protection from apoptosis was found to be dependent on reactive oxygen species (ROS) generation [148]; in addition, ROS is known to modulate vital physiological processes including cell growth, angiogenesis, contraction, and relaxation of VSM [150]. 20-HETE was found to increase both superoxide and NO production in BPAECs resulting in promotion of angiogenesis [148,149,150,151]. In addition, 20-HETE maintained the stability of mitochondria membrane and relieved the activation of caspase-9 and caspase-3 [149]. 5,14,20-HEDE and 5,14,20-HEDGE enhanced ROS production in endothelial cell in intact lung ex vivo as well as in cultured pulmonary artery endothelial cells [146]. 

### 4.3. Role of 20-HETE in Pulmonary Vascular Tone

20-HETE alters vascular tone signaling in the pulmonary vasculature and plays a role in VSM remodeling [146,147,149,150]. Unlike its constrictor effects on systemic circulation, peripheral arteries, cerebral and renal vessels, 20-HETE is an endothelial-dependent dilator of pulmonary arteries [141,152,153]. 20-HETE relaxed contractile responses to 5-hydroxytryptamine concentration-dependently (0.01–10 microM) in fresh and 1 day culture of pulmonary arteries [154]. 20-HETE was found to induce a dose-dependent vasodilation of isolated human small pulmonary arteries which was inhibited by indomethacin [155], suggesting that 20-HETE could act as a COX-dependent vasodilator for human pulmonary arteries [156]. 20-HETE-dependent pulmonary arteries vasodilation is mediated by increasing intracellular Ca^2+^ concentration and NO release and found to be endothelial-dependent [152,157]. 20-HETE also contributes to vascular endothelial growth factor (VEGF)-stimulated NO release [157,158]; VEGF-induced NO release was attenuated by pretreating the BPAECs with DDMS as well as HET0016 [157]. The potent dilatory response to 20-HETE in human and rabbit pulmonary vascular and bronchiole rings was found to be dependent on an intact endothelium and COX enzyme [93,143]. Human lung microsomes were able to convert 20-HETE into prostanoids suggesting that COX in vascular tissue metabolizes 20-HETE to a vasodilatory compound [155]. Of interest, CYP4 has a unique expression in BPAECs versus systemic arterial endothelial cells suggesting that CYP4 could be an important mediator of endothelial-dependent vasoreactivity in pulmonary arteries [157]. Moreover, a recent study showed that 20-HETE, through gap junctions, appears to modulate smooth muscle myosin heavy chain expression and contribute to the sustained state of hypoxic pulmonary vasoconstriction development [159].

Interestingly, 20-HETE was demonstrated to be more potent vasoconstrictors than phenylephrine and KCl on small pulmonary arteries of rat and did not exhibit any relaxant effects on pulmonary artery rings precontracted with phenylephrine. Potency of 20-HETE as a vasoconstrictor in small pulmonary arteries was attenuated in pulmonary artery rings from lung with pneumonia [156]. It seems that 20-HETE has varied vasoactive effects dependent on the vascular beds studied, where vasoactive effects of 20-HETE can be influenced by the activity of other enzyme systems such as NOS and COX [156]. In sheep pulmonary artery, 20-HETE has a predominant role in the inhibition of vascular Na^+^-K^+^-ATPase activity, where it significantly attenuated KCl-induced relaxations. In addition, AA caused concentration-dependent inhibition of KCl-induced relaxations and increases basal arterial tone in pulmonary vasculature in sheep, an effect which was completely reversed by 17-ODYA as well as HET0016 [160]. Also in contrast to its action in adult pulmonary circulation, 20-HETE causes constriction in newborn pulmonary resistance-level arteries at resting tone [153] and was found to be released in fetal pulmonary circulation in response to acute increases in pulmonary artery pressure resulting in vasoconstriction [161]. 20-HETE as a vasoconstrictor, causes blockade of Ca^2+^-activated K^+^ (K_Ca_) channels and inhibits the formation of NO [93]. Inhibition of 20-HETE might have a therapeutic role in neonatal conditions characterized by pulmonary hypertension; DDMS was found to abolish the vasoconstrictor response to ductus arteriosus compression in the presence of nitro-l-arginine (l-NA; to inhibit shear-stress vasodilation) [161].

### 4.4. Role of 20-HETE in the Airways

In guinea pigs, airway smooth muscle (ASM) cells were found to produce 20-HETE which acted as a bronchoconstrictor and induced concentration-dependent tonic responses [94,162]. However, in human ASM, 20-HETE causes a concentration-dependent relaxation in bronchi precontracted with methacholine or AA [163]. Similarly, 20-HETE produced a concentration-dependent relaxation of rabbit bronchial rings precontracted with KCl or histamine [164]. Differences between observations regarding 20-HETE effect on ASM are likely related to inter-species differences [163]. In rabbit, 20-HETE modulates airway resistance in a COX-dependent manner; 20-HETE-dependent relaxation of bronchial rings was blocked by indomethacin suggesting that epithelial COX converts 20-HETE to a bronchial relaxant which elicited relaxation of bronchial rings [164]. However, in human bronchi, the responses to 20-HETE were not modified by indomethacin pretreatments. Moreover, 20-OH-PGE_2_ had basically no relaxing effect on bronchi precontracted with methacholine chloride, suggesting that the relaxing effect induced by 20-HETE was not related to prostanoid formation [163]. 20-HETE-dependent hyperpolarization and controlled relaxation of ASM in human distal bronchi depends on activation of large conductance K_Ca_ channels [163]. Interestingly, 20-HETE seems to play an important role in mediating acute ozone-induced airway hyper-responsiveness responsible for increasing morbidity and mortality in patients with obstructive airway diseases and asthma [165]. Interestingly, a recent study performed in Balb/c mice showed that local administration of 20-HETE led to proinflammatory action, associated with airway neutrophilia and macrophage activation, thus contributing to airway hyperresponsiveness [166].

## 5. Role of 20-HETE in the Brain

### 5.1. Formation, Metabolism and Regulation of 20-HETE in the Brain

Human CYP2U1 metabolizes AA exclusively to 19-HETE and 20-HETE with abundant transcripts in cerebellum suggesting an important role in fatty acid signaling processes in brain [167]. Brain astrocytes synthesize and release 20-HETE which acts as cerebral arterial myocyte constrictor [168]. In addition, cerebral microvessels convert 20-HETE to 20-COOH-AA which produces vasoconstriction in these vessels, this reaction was found to be catalyzed by alcohol dehydrogenase (ADH), mainly ADH_4_ [169]. As for regulation, PaCO_2_ is an important factor in the regulation of cerebral circulation; changes in CO_2_ concentration could cause changes in cerebral blood flow. CO_2_ can decrease the expression of brain CYP4A during hypercapnia and increase its expression during hypocapnia, suggesting that 20-HETE plays an important role in CO_2-_mediated cerebrovascular reactivity [170]. NO was also found to affect 20-HETE synthesis by decreasing its level and inhibiting its formation [171]. 

### 5.2. Role of 20-HETE in Regulating Vascular Tone in Brain

Brain does not store glycogen and thus requires a constant supply of glucose and oxygen for the continuous demands of cerebral function [172]; hence, the control of cerebral vessel diameter and cerebral blood flow is of fundamental importance in maintaining healthy brain function [173]. 20-HETE has significant role in the regulation of vascular tone, autoregulation of cerebral blood flow, and the neurovascular coupling (coupling of regional brain blood flow to neuronal activity) [174,175,176]. 20-HETE was found to be generated in various cell types in the brain and cerebral blood vessels [174,177]. Glial cells, non-neuronal cells, were found to play an important role in mediating neurovascular coupling by inducing the production of EETs and 20-HETE, which dilate and constrict vessels, respectively [178,179]. Recent studies have shown that astrocytes, characteristic star-shaped glial cells, are critical players in the regulation of cerebral blood vessel diameter; astrocytes produce 20-HETE from AA [173]. Furthermore, 20-HETE has been implicated in arteriolar constriction during astrocyte activation in brain slices, and modulates vasodilation in cerebral cortex during sensory activation [171]. 20-HETE synthesis limits the duration of the response to prolonged activation of Group I metabotropic glutamate receptors (mGluR) on astrocytes. Under normal conditions in vivo stimulation of mGluR increases cerebral blood flow [180]. Protein kinase C was found to be an integral part of the signal transduction pathway by which 20-HETE elicits vasoconstriction of cerebral arteries and inhibits the whole cell K^+^ current in cat cerebral VSM [181]. 20-HETE also activates l-type calcium current in cerebral arterial smooth muscles [78]. Interestingly, it was found that 20-HETE is an important contributor to cerebral vasoconstriction associated with the onset of ventilation at birth [182]. Recent studies showed that 20-HETE-dependent vasoconstriction in the cerebellum is mediated by metabotropic glutamate receptors [183]. Moreover, 20-HETE induces cerebral parenchymal arteriolar constriction via superoxide production resulting from NADPH oxidase activation, and propofol could prevent this constriction via inhibiting NADPH oxidase [184]. Similarly, 20-HETE inhibitors were found to be associated with a decrease in superoxide production and activation of caspase-3 [185]. Although vasodilation of the brain blood vessels is known to be mediated by prostaglandin E2, nitric oxide production is also needed to suppress 20-HETE formation [186]. Of interest, 20-HETE could dilate mouse basilar artery preconstricted with U-46619, a thromboxane mimetic, in vitro. This action was inhibited by indomethacin suggesting a COX-dependent mechanism. In fact, mouse brain endothelial cells were found to convert 20-HETE to 20-OH-PGE_2_, which was as potent as PGE_2_ in dilating the basilar artery [187].

### 5.3. Role of 20-HETE in Cerebral Ischemia/Reperfusion (I/R) Injury

20-HETE is a potent vasoconstrictor of cerebral microvessels which contributes to I/R injury [188,189,190,191] being a mediator of free radical formation and tissue death [119]. 20-HETE plays an important role in the development of neurological and functional deficits, and contributes to infarct size after focal cerebral ischemia [192,193]. It also contributes to neurodegeneration after global cerebral ischemia in immature brain [193]. In rat subarachnoid hemorrhage (SAH) model, 20-HETE formation was found to be substantially elevated in the cerebrospinal fluid (CSF) with a concomitant 30% decrease in cerebral blood flow [189,194,195]. Similar data was obtained in aneurysmal subarachnoid hemorrhage (aSAH) model; 20-HETE was reported to affect cerebral microvascular tone and cerebral blood flow [196]. 20-HETE levels in CSF were also associated with delayed cerebral ischemia, neurological decline, and long-term functional and neuropsychological impairment [196]. GG genotype and G allele of CYP4F2 was associated with ischemic stroke in the male Northern Chinese Han population [197]. Interestingly, 20-HETE was present at physiologically-relevant concentrations in CSF of SAH patients [189]. 

Inhibitors of 20-HETE synthesis reduced infarct volume and decreased cerebral damage after cerebral ischemia and after I/R in brain without altering blood flow providing direct neuroprotective actions [174,185,198]. In vivo, DDMS and 6,15,20-HEDE were found to attenuate autoregulation of cerebral blood flow to elevation of arterial pressure [175]. HET0016 administration reduced brain damage in a rat model of thromboembolic stroke [199] and it protects blood brain barrier dysfunction after I/R through regulating the expression of MMP-9 and tight junction proteins. Moreover, this protection may be due to inhibition of oxidative stress and JNK pathway [200]. Also, rats treated with HET0016, 90 min prior to temporary middle cerebral artery occlusion, showed 79.6% reduction in 20-HETE in the cortex, pronounced reduction in lesion volume and attenuation of the decrease in cerebral blood flow [191]. 6,15,20-HEDE showed similar effects to HET0016 in reducing infarct size after transient occlusion of the middle cerebral artery [198]. In neonatal piglets, HET0016 administration after hypoxia-ischemia improved early neurological recovery and protected neurons in putamen [193]. Blockade of the synthesis of 20-HETE with TS-011 opposed cerebral vasospasm and reversed the fall in cerebral blood flow following SAH. In addition, it reduced infarct size and improved neurological deficits in ischemic models of stroke [188,201,202]. TS-011 reduced infarct volume in rats after transient occlusion of the middle cerebral artery and reduced neurological deficits as well [198,202]. When given in combination with tissue plasminogen activator, TS-011 improved neurological outcomes in the stroke model in monkey [202]. Thus, TS-011 may provide benefits in patients suffering ischemic stroke [192]. In vitro application of DDMS, or 6,15,20-HEDE eliminated pressure-induced constriction of rat middle cerebral arteries [175]. Organotypic hippocampal slice cultures subjected to oxygen-glucose deprivation (OGD) and reoxygenation showed 2-fold increase in 20-HETE production, however inhibition of 20-HETE synthesis by HET0016 or action by 6,15,20-HEDE reduced the cell death. On the other hand, administration of 5,14,20-HEDE increased injury after OGD [185]. 

## 6. Role of 20-HETE in Tumors

**Figure 6:**
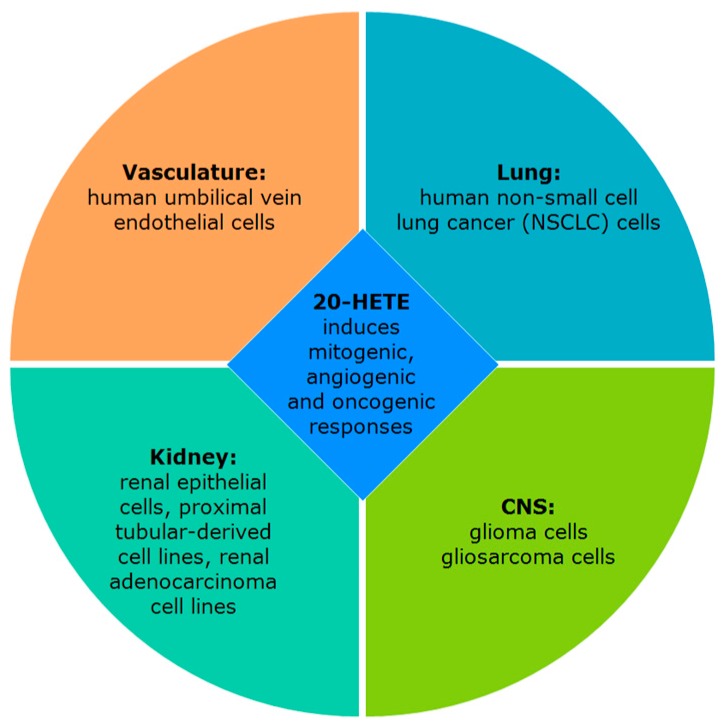
20-HETE role in carcinogenicity.

Increased generation of 20-HETE was reported to induce mitogenic and angiogenic responses both in vitro and in vivo (summarized in Figure 6) [203,204]. 

In human umbilical vein endothelial cells, administration of 5,14,20-HEDE induced mitogenesis, whereas HET0016 abolished the mitogenic response to VEGF [205]. 20-HETE is also considered a mitogen for smooth muscle cells [43]. 5,14,20-HEDE induced angiogenesis in rat cornea, whereas HET0016 inhibited growth factor-induced angiogenesis by 80%–90% [205]. In lung, 20-HETE was found to promote tumor angiogenesis and metastasis in human non-small cell lung cancer (NSCLC) cells. In vitro, addition of 5,14,20-HEDE or transfection of A549 cells, a human NSCLC cell line, with CYP4A11 expression vector significantly induced invasion, whereas treatment with HET0016 or 6,15,20-HEDE inhibited invasion. In vivo, CYP4A11 transfection significantly increased tumor weight, microvessel density, and lung metastasis, whereas HET0016 or 6,15,20-HEDE administration decreased tumor volume, microvessel density, and spontaneous pulmonary metastasis occurrences [206]. In kidney, 20-HETE has been reported to promote mitogenicity and proliferation in renal epithelial cells [207,208]. In vitro, 20-HETE was found to be a potent mitogen to swine proximal tubular-derived cell lines, LLC-PK1 and opossum kidney (OK) [209]. However no mitogenic effects were observed with 20-HETE in LLCPKcl4, a subclone from the parental LLC-PK1 cells [210]. HET0016 and 6,15,20-HEDE inhibited the proliferation of 786-O and 769-P, human renal adenocarcinoma cell lines, while having little effect on the proliferation of primary cultures of normal human proximal tubule epithelial cells. In vivo, 6,15,20-HEDE administered daily to athymic nude mice implanted subcutaneously with 786-O cells, reduced the growth of the tumors by 84% [207]. In benign prostatic hypertrophy and prostate cancer patients, urinary concentrations of 20-HETE were significantly higher than normal subjects and it was decreased to normal concentrations after removal of prostate gland [211]. Moreover, Vanella et al. showed that ellagic acid (an antioxidant) mediates a new pathway for its chemotherapeutic property, other than its anti-proliferative and pro-differentiation properties, through a mechanism that involves a decrease in 20-HETE synthesis in prostate cancer cells [212]. In human mammary carcinoma, CYP4Z1 which considered a novel CYP4 is over-expressed and was found to be associated with high-grade tumors and poor prognosis, in addition, stable expression of CYP4Z1 in T47D human breast cancer cells lead to higher contents of 20-HETE, whereas HET0016 potently inhibited the tumor-induced angiogenesis with associated changes in the intracellular levels of 20-HETE [213]. Additionally, HET0016 inhibits pro-angiogenic factors and ceases growth of triple negative breast cancer in mice [214].

Similarly, samples from thyroid, breast, colon, and ovarian cancer revealed that expression of CYP4A/4F genes is highly elevated in cancer samples as compared to matched normal tissues [204]. As for CNS, 20-HETE was found to have proto-oncogenic properties in U251 human glioma cells, as pretreatment with exogenous 20-HETE and 5,14,20-HEDE increased U251 cell proliferation [215,216]. Transfection of U251 cells with CYP4A1 cDNA increased the formation of 20-HETE and increased their proliferation rate by 2-fold, whereas HET0016, small interfering RNA (siRNA) against the enzyme, and 6,15,20-HEDE abolished this hyperproliferation [215]. In vivo, implantation of CYP4A1 transfected U251 cells in the brain of nude rats resulted in a 10-fold larger tumor volume after ten days [215]. HET0016 inhibited U251 basal cell proliferation and decreased its induced angiogenesis [216]. Similar results obtained in 9L rat gliosarcoma cells; 5,14,20-HEDE increased its proliferation in vitro, while HET0016 achieved 55% reduction in proliferation after 48 h of incubation. In vivo, chronic administration of HET0016 for two weeks reduced the volume of 9L tumors by 80%, and increased mean survival time of the animals [217]. Interestingly, although both CYP4A mRNA and protein were detected in U251, it did not synthesize 20-HETE in the presence of AA suggesting that HET0016 suppresses U251 proliferation in vitro by mechanisms that may involve activities other than inhibiting 20-HETE synthesis [215,216]. HET0016 is also able to inhibit tumor growth and migration depending on the schedule of drug administration. The addition of HET0016 to vatalanib (a small molecule protein kinase inhibitor that inhibits angiogenesis) may mitigate the undesired effect of vatalanib [218]. Similarly, neither 9L cells grown in vitro nor 9L tumors removed produced 20-HETE when incubated with AA; however, the normal surrounding brain tissue avidly makes 20-HETE. This data suggests that HET0016 may act in vivo by inhibiting the formation of 20-HETE by the surrounding tissue, whereas its antiproliferative effects in vitro seems to be unrelated to its ability to inhibit the formation of 20-HETE [216,217]. In traditional Chinese medicine, Scolopendra subspinipes mutilans L. Koch has been used for cancer treatment for hundreds of years. Scolopendra Polysaccharide-Protein Complex (SPPC) significantly inhibited the growth of S180, mouse sarcoma cell line, transplanted in mice. SPPC prolonged the survival time of H22, mouse hepatoma cell line, bearing mice. SPPC diminished CYP4A and 20-HETE in tumor-associated macrophages [219].

## 7. Role of 20-HETE in Metabolic Syndrome and Diabetes

Mice fed high-fat diet had significantly higher 20-HETE/(EET+DHET) formation rate ratio in the liver and kidney, suggesting a role in pathogenesis and progression of metabolic syndrome [220]. Moreover, increased levels of 20-HETE significantly disrupt survival and function of endothelial cells in metabolic syndrome leading to impaired coronary collateral growth, hence 20-HETE antagonists could be a potential therapeutic choice in this case [221]. In vitro, 20-HETE has been shown to be involved in adipogenesis. Kim et al. examined the effect of exogenous 20-HETE on mesenchymal stem cell-derived adipocytes and concluded that 20-HETE increased adipogenesis in a concentration-dependent manner [222]. In vivo, 20-HETE contributes to the elevation in vascular reactivity in diabetic animals [223], and was reported to play a role in diabetes-enhanced vasoconstriction of mesenteric and renal vessels [224]. 20-HETE is also involved in the early decrease of retinal hemodynamics in diabetic mice which could be attenuated by HET0016 [224]. Chronic treatment of the diabetic rats with ABT or HET0016 attenuated the responses to vasoconstrictors, norepinephrine, ET-1, and AngII, in mesenteric vascular bed and renal artery segments [223]. Eid et al. suggested that 20-HETE inhibitors could have a pivotal therapeutic potential in the alleviation of diabetic nephropathy [225]. Moreover, hyperglycemia decreases 20-HETE production in the glomeruli suggesting an important role in glomerular damage in the early stage of diabetic nephropathy. Rats treated with streptozotocin for five weeks, to induce diabetes, had a significant decrease in glomerular CYP4A expression; however, when they were treated with clofibrate, glomerular CYP4A expression and 20-HETE production were induced while urinary protein excretion was reduced [37]. Isolated perfused kidney from rats with streptozotocin-induced diabetes show great reduction in 20-HETE release, whereas insulin treatment for two weeks reverses hyperglycemia and renal deficiency in 20-HETE [226].

## 8. Summary and Conclusions

The formation of 20-HETE has been demonstrated to be through different members of the CYP ω-hydroxylase enzymes, the CYP4A, CYP4F and CYP2U subfamilies. However, the predominant form responsible for 20-HETE formation differs from organ to organ, from species to species and from males to females. For example, Cyp4a12a is the predominant 20-HETE forming enzyme in the mouse kidney and its expression levels dictates the sex and strain specific differences in 20-HETE formation and may explain sex and strain differences in susceptibility to hypertension and target organ damage [48]. In female mice compared to male mice, low AA hydroxylase activity was observed in the kidney and this was associated with very low Cyp4a12a mRNA and protein expression levels, despite high Cyp4a10 and Cyp4a14 expression levels [48]. Thus, these observations highlight the importance of determining the expression of each CYP ω-hydroxylase isoform in different organs.

Although it has been previously reported that 20-HETE plays a negative role in the heart and vasculature [2,3,90,227], it has been demonstrated to have a protective effect in the kidney. 20-HETE plays an anti-hypertensive effect through its regulation of sodium reabsorption in the proximal tubule and TAL [21,38,41,47,48,61,70]. This effect protects the cardiovascular system from salt-retention hypertension, however 20-HETE is a potent constrictor produced by the preglomerular vasculature and could participate in the development of hypertension [40,43,56,71]. Hence it is obvious that a renal 20-HETE level is a double-edged sword, depending on its levels in various locations within the nephron. Moreover, 20-HETE plays a crucial role in renal I/R injury in addition to polycystic kidney diseases. Thus specific targeting of 20-HETE production and/or action might serve as a treatment strategy in renal diseases in the future.

In the liver, the role of 20-HETE is still to be determined although it represents 50%–75% of CYP-dependent AA metabolites in this organ [93,133]. Importantly, it has been demonstrated that 20-HETE is a potent activator of PPAR_α_ and is supposed to exert important functions in lipid homeostasis and in controlling fat-dependent energy supply and metabolism [135,136]. On the other hand, 20-HETE levels have been shown to be elevated in liver pathophysiological disease states such as cirrhotic ascites [139]. Therefore, future studies investigating the physiological and pathophysiological role of hepatic 20-HETE will be of great importance.

The protective effect of 20-HETE is not only limited to the kidney, but it has been also demonstrated in the lung. For example, 20-HETE mediates cell survival signaling in pulmonary vasculature [146,147] and was found to protect against apoptosis in bovine pulmonary artery endothelial cells [148,149]. Moreover, 20-HETE was found to dilate the pulmonary artery in contrast to its vasoconstrictive effect in systemic circulation, peripheral arteries, cerebral and renal vessels [141,152,153]. The controversy between the vasodilator effect of 20-HETE in the lung and its vasoconstrictor effect in other vasculature could be attributed to a COX-dependent transformation of 20-HETE to metabolites possessing vasodilator effects (20-OH-PGI_2_, and 20-OH-PGE_2_) [156]. However, this postulation requires further studies to uncover the exact mechanism.

Unlike the other organs, brain metabolizes AA to 20-HETE mainly through CYP2U1 [167], which is abundant in the cerebellum, implicating the importance of this enzyme in 20-HETE mediated fatty acid signaling processes. This is not the only difference between brain and the other organs. That is, brain does not store glycogen [172] as a reservoir of energy and thus requires continuous supply of glucose and oxygen. In this regard, 20-HETE rises as a crucial modulator of brain vascular tone, cerebral blood flow, and neurovascular coupling. Furthermore, 20-HETE is a potent vasoconstrictor for cerebral microvessels contributing to ischemia reperfusion injury [188,189,190,191]. Altogether, maintaining homeostatic levels of 20-HETE is pivotal in determining brain functions and altering its levels might contribute to brain pathophysiological disease states.

Of interest, although 20-HETE through its vasoconstrictor effect might appear at the first glance as a tumor suppressor, recent studies have shown that 20-HETE promotes tumor formation through promoting angiogenesis and mitogenesis. Thus targeting its production and/or action might serve as a treatment strategy in carcinogenesis.

Further studies are needed to investigate the exact events and mechanisms associated with different 20-HETE levels in vital organs. Our success in understanding these events would help in rational designing of therapeutic strategies to reduce 20-HETE-mediated disorders.

## Abbreviations

10-SUYS: acetylenic fatty acid sodium 10-undecynyl sulfate
17-ODYA: 17-octadecynoic acid
20-COOH-AA: 20-carboxyeicosatetraenoic acid
20-HETE: 20-hydroxyeicosatetraenoic acid
20-OH PGH2: hydroxyl analogue of vasoconstrictor prostaglandin H2
5,14,20-HEDE; WIT003: 20-hydroxyeicosa-5(Z),14(Z)-dienoic acid
5,14,20-HEDGE: *N*-[20-hydroxyeicosa-5(Z),14(Z)-dienoyl]glycine
6,15,20-HEDE; WIT002: 20-hydroxyeicosa-6(Z),15(Z)-dienoic acid
6,15,20-HEDGE: 20-hydroxyeicosa-6(Z),15(Z)-dienoyl]glycine
AA: Arachidonic acid
ABT: 1-Aminobenzotriazole
ADH: alcohol dehydrogenase
AngII: vasoconstrictors Angiotensin II
aSAH: aneurysmal subarachnoid hemorrhage model
ASM: airway smooth muscle cells
BPAECs: bovine pulmonary artery endothelial cells
CMV: cyto-megalovirus
CO: carbon monoxide
CoCl_2_: Cobalt(II) chloride
COX: cyclooxygenase
CSF: cerebrospinal fluid
CYP: cytochrome P450
DBDD: dibromododec-11-enoic acid
DDMS: *N*-methylsulfonyl-12,12-dibromododec-11-enamide
EETs: epoxyeicosatrienoic acids
eNOS: Endothelial NOS
ET: endothelin
EGFR: epidermal growth factor receptor
HET0016: *N*-hydroxy-*N’*-(4-butyl-2methylphenyl)formamidine
HETEs: hydroxyeicosatetraenoic acids
HO: heme oxygenase enzymes
I/R: Ischemia/Reperfusion injury
KAP: kidney androgen-regulated protein
K_Ca_: Ca^2+^-activated K^+^ channels
l-NA: nitro-l-arginine
l-NAME: N(ω)-nitro-l-arginine-methyl ester
LOX: lipoxygenase
LTs: leukotrienes
LXs: lipoxins
mGluR: Group I metabotropic glutamate receptors
NOS: NO synthase
NSCLC: non-small cell lung cancer cells
OGD: oxygen-glucose deprivation
Palb: albumin permeability
PAs: pulmonary arteries
PGs: prostaglandins
PKC: protein kinase C
PPARα: Peroxisome proliferator-activated receptor α
ROS: reactive oxygen species
RUPP: reductions in uterine perfusion pressure
RVR: renal vascular resistance
SAH: subarachnoid hemorrhage model
SD: Sprague Dawley
sEH: soluble epoxide hydrolase
siRNA: small interfering RNA
SPPC: Scolopendra Polysaccharide-Protein Complex
TAL: thick ascending limb of the loop of Henle
TGF-β: transforming growth factor-beta
TS011: *N*-(3-Chloro-4-morpholin-4-yl)Phenyl-*N’*-hydroxyimido formamide
VEGF: vascular endothelial growth factor
VSM: vascular smooth muscle
